# Microcystin-Leucine Arginine Causes Cytotoxic Effects in Sertoli Cells Resulting in Reproductive Dysfunction in Male Mice

**DOI:** 10.1038/srep39238

**Published:** 2016-12-15

**Authors:** Yabing Chen, Yuan Zhou, Jing Wang, Lihui Wang, Zou Xiang, Dongmei Li, Xiaodong Han

**Affiliations:** 1 Immunology and Reproduction Biology Laboratory & State Key Laboratory of Analytical Chemistry for Life Science, Medical School, Nanjing University, Nanjing, 210093, China; 2 Jiangsu Key Laboratory of Molecular Medicine, Nanjing University, Nanjing, 210093, China; 3 School of Basic Medicine, Nanjing University of Chinese Medicine, Nanjing, 210093, China; 4 Department of Health Technology and Informatics, Faculty of Health and Social Sciences, The Hong Kong Polytechnic University, Hung Hom, Kowloon, Hong Kong, China

## Abstract

Microcystin-leucine arginine (MC-LR) is a potent toxin for Sertoli cells. However, the specific molecular mechanisms of MC-induced cytotoxicity still remain unclear. In this study, we performed a comprehensive analyses of changes of miRNAs and mRNAs in Sertoli cells treated with MC-LR. Through computational approaches, we showed the pivotal roles of differentially expressed miRNAs that were associated with cell metabolism, cellular growth and proliferation, cell-to-cell signaling and interaction and cellular movement. Ingenuity Pathway Analyses (IPA) revealed some differentially expressed miRNAs and mRNAs that may cause reproductive system diseases. Target gene analyses suggested that destruction in tight junctions (TJ) and adherens junctions (AJ) in testes may be mediated by miRNAs. Consistent with a significant enrichment of chemokine signaling pathways, we observed numerous macrophages in the testes of mice following treatment with MC-LR, which may cause testicular inflammation. Moreover, miR-98-5p and miR-758 were predicted to bind the 3′-UTR region of the mitogen-activated protein kinase 11 (MAPK11, p38 β isoform) gene which stimulates tumor necrosis factor-α (TNF-α) expression in Sertoli cells. TNF-α could interact with the tumor necrosis factor receptor 1 (TNFR1) on germ cells leading to induction of germ cell apoptosis. Collectively, our integrated miRNA/mRNA analyses provided a molecular paradigm, which was experimentally validated, for understanding MC-LR-induced cytotoxicity.

Microcystins (MCs) are a family of cyclic heptapeptide cytotoxins produced and released by several genera of freshwater cyanobacteria. With the frequent outbreaks of cyanobacterial blooms, an increasing number of lakes and rivers are facing the threat of MC pollution. As MCs can enter the body of all the living creatures through drinking water, they may pose a substantial health hazard to humans higher up in the food chain owing to enrichment of MCs in aquatic creatures^1^. Previous reports have identified the potential of MCs to cause hepatotoxicity, neurotoxicity, kidney impairment, and gastrointestinal disorders^2^,^3^,^4^,^5^. In view of the biological toxicity of MCs, the World Health Organization (WHO) set an upper limit of 1 μg/L MCs in freshwater. Alarmingly, studies from various countries revealed that the concentrations of MCs in some natural water bodies are much higher. The concentration of MCs in Lake Taihu, China, was reported to reach 15.6 μg/L in summer^6^. Moreover, MCs with varying concentrations from 10 to 500 μg/L were also detected in eutrophic lakes in America^7^. Up to date, more than 100 MC variants have been examined, among which MC-leucine arginine (MC-LR) is the most abundant and the most toxic MC, comprising 46–99.8% of the total MCs in the natural waters^8^. Our previous studies have identified that gonads are important target organs of MC-LR. Acute, sub-acute and chronic low-dose exposures to MC-LR all cause toxic effects on the male reproductive system in rats^9^,^10^. Decreased testosterone levels, testicular atrophy, declines of sperm concentrations, and high incidences of sperm abnormality were also observed in rats following exposure to chronic low-dose MC-LR^9^. Furthermore, we also found that MC-LR may exert its toxicity on cultured germ cells and Sertoli cells *in vitro* resulting in reduced cell viability^11^,^12^,^13^,^14^.

Testicular Sertoli cells play important roles in spermatogenesis as they nourish sperm cells and contribute to the formation of the blood-testis barrier (BTB) that depends on the existence of Sertoli-Sertoli cell tight junctions^15^. Our recent studies suggest that MC-LR can enter Sertoli cells and induce autophagy and apoptosis in Sertoli cells *in vitro*^11^,^16^. However, the underlying molecular mechanisms associated with Sertoli cell toxicity induced by MC-LR remain unclear.

miRNAs are small (~21 nucleotides) non-coding RNAs which can bind to the complementary regions in the mRNA molecules and then degrade target mRNAs or repress their translation^17^. They are expressed in a wide range of tissues in many species, and computational predications indicate that more than one third of all human genes may be miRNA targets^17^. As an important regulatory factor, miRNA regulates diverse cellular processes, including the proliferation, apoptosis, and responses to various stimuli^13^,^18^,^19^. Therefore, identification and characterization of the changes of miRNAs can reveal the dynamic regulation of cellular functions.

In order to decipher the upstream regulatory networks associated with Sertoli cell cytotoxicity induced by MC-LR, here we took the advantage of bioinformatics technology to present an integrative analyses of the whole gene expression pattern and its regulatory miRNA networks. The interacting networks revealed key miRNA/mRNA interacting pairs that are correlated with cell apoptosis, tight junction (TJ) and adhere junction (AJ) destruction, and up-regulation of tumor necrosis factor-α (TNF-α) expression in Sertoli cells. To further identify the miRNA/mRNA regulatory network, we conducted both *in vivo* and *in vitro* experiments. We observed that exposure to MC-LR caused BTB destruction, massive Sertoli cell and germ cell apoptosis, testicular inflammation, and autoantibody generation, resulting in oligospermia. Taken together, our integrative miRNA/mRNA analyses has provided a valuable tool for understanding effectively complex signaling networks associated with reproductive dysfunction induced by MC-LR.

## Results

### MC-LR modulates miRNA profiles in Sertoli cells

To confirm miRNA microarray data^20^, we assessed the expression of 10 miRNAs by quantitative PCR (q-PCR) (Supplementary Table S1). The data generated by the q-PCR assay were consistent with the microarray analyses, and the correlation-coefficient between the mean values of ten individuals generated by both techniques for each miRNA was statistically significant (Supplementary Figure S1A and Supplementary Table S1), indicating the reliability of the array data generated by miRNA microarray. In this study, many miRNAs associated with azoospermia, such as miR-199a-5p^21^, miR-181a^22^, miR-221^23^, miR-141^19^, and miR-429^19^,^24^, were found to be significantly modulated by exposure to MC-LR (Table 1). Moreover, some miRNAs involved in the mechanisms of other reproductive system diseases, including the urinary tract tumor, prostate cancer, and genital tumor, were also detected^25^,^26^,^27^,^28^.

### MC-LR induces differentially expressed mRNAs with possible molecular functions

**Figure 1:**
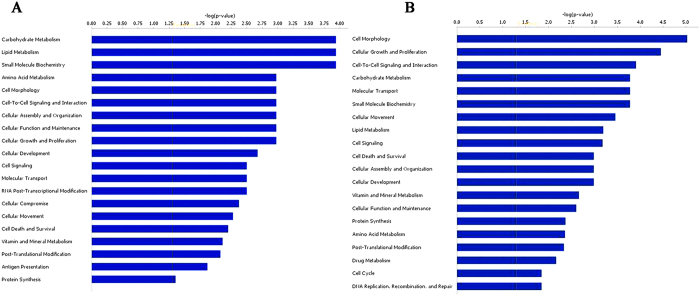
Functional analysis of the differentially expressed mRNA in Sertoli cells following exposure to 500 nm Microcystin-leucine arginine (MC-LR). (**A**) Gene Ontology (GO) analysis of the differentially expressed genes was performed. The top 20 enriched GO terms describing the molecular functions were listed. (**B**) GO enrichment analysis of target genes of the differentially expressed miRNAs in Sertoli cells in response to MC-LR stress was performed and the results were listed.

We selected six genes for q-PCR validation; our results demonstrate that their expression changes were consistent with the microarray data (Supplementary Figure S1B). Categorizing the altered genes may facilitate the understanding of the biological processes regulated by differentially expressed genes in Sertoli cells. Gene Ontology (GO) enrichment analyses revealed that these genes with aberrant expression levels take part in many biological processes, including cell metabolism (e.g. carbohydrate metabolism, lipid metabolism, vitamin and mineral metabolism, and amino acid metabolism), cellular growth and proliferation, cell-to-cell signaling and interaction, cellular movement, gene expression, and the macromolecule biosynthetic process (Fig. 1A). Pathway enrichment analyses showed that communication between innate and adaptive immune cells, the p53 signaling pathway, tumor necrosis factor receptor 1 (TNFR1) signaling, and tight junction-related pathways were significantly enriched (Table 2).

### Effects of MC-LR on proto-oncogene expression in Sertoli cells and testes

**Figure 2:**
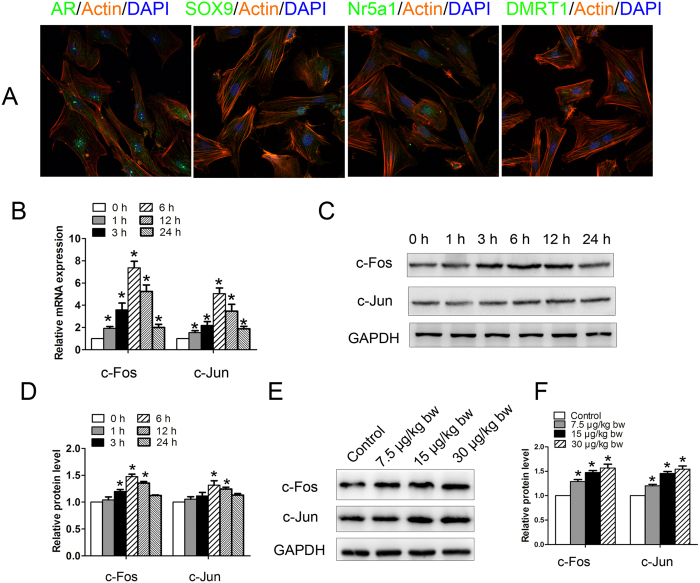
Induction of proto-oncogene expression in Sertoli cells and testes by microcystin-leucine arginine (MC-LR). **(A**) Mouse Sertoli cells were collected and cultured for 2 days. Representative immunofluorescence analyses of the marker proteins of Sertoli cells are shown (×600). Nuclei were stained with DAPI (blue), and phalloidin (orange) was used to label the actin. Sertoli cells were treated with 500 nm MC-LR for various durations as indicated. (**B**) Changes of c-Fos and c-Jun transcript were examined by q-PCR, and relative mRNA abundance was calibrated based on the mRNA amount of the corresponding gene in the untreated group (mean ± SD. *P < 0.05, compared with the control). (**C**,**D**) Expression of c-Fos and c-Jun in Sertoli cells was detected by western blotting. Representative gel electrophoresis bands are shown (**C**), and the expression levels of the proteins were quantified by densitometry and normalized to the expression of GAPDH (**D**). Densitometry data are shown as mean ± SD. *P < 0.05, compared with the control. Male mice were injected intraperitoneally with saline or MC-LR (7.5 μg/kg body weight (bw), 15 μg/kg bw, and 30 μg/kg bw) for 7 days. (**E**,**F**) The expression levels of c-Fos and c-Jun were analyzed by western blotting. Representative gel electrophoresis bands are shown (**E**), and the expression levels of the proteins were quantified by densitometry and normalized to the expression of GAPDH (**F**). Densitometry data are shown as mean ± SD. *P < 0.05, compared with the control.

Immunofluorescence assay was used to identify cultured Sertoli cells. Cultured Sertoli cells were consistently positive for androgen receptor (AR), sex-determining region Y box 9 (SOX9), nuclear receptor subfamily 5, group A, member 1 (Nr5a1), and doublesex-related transcription factor 1 (DMRT1), suggesting a high purity of our cultured cells (Fig. 2A). We examined c-Fos and c-Jun mRNA abundance in Sertoli cells following exposure to MC-LR for 1, 3, 6, 12, and 24 h by q-PCR. As shown in Fig. 2B, the mRNA levels of c-Fos and c-Jun increased immediately after 1 h of MC-LR treatment, and reached a peak at 6 h post-exposure (up to 8-fold and 5-fold for c-Fos and c-Jun, respectively. In contrast to early alteration of mRNA levels, protein expression of c-Fos and c-Jun was not significantly elevated until 6 h (Fig. 2C,D). Moreover, relatively moderate changes of their protein levels were observed compared to their mRNA abundance. Similarly, MC-LR treatment also induced c-Fos and c-Jun protein expression in testes (Fig. 2E,F).

### Integrated miRNA/mRNA analyses reveal regulatory networks for differentially expressed miRNAs

**Figure 3:**
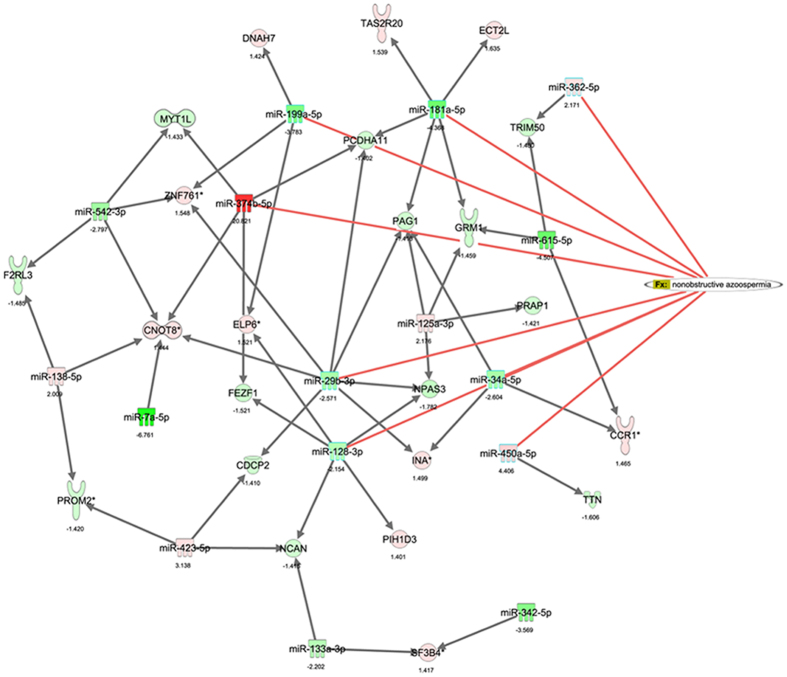
The miRNA regulatory networks and their target genes involved in nonobstructive azoospermia. Ingenuity pathway analysis (IPA) was performed to describe functional relationships among miRNAs and genes based on the known associations in the databases, and some differentially expressed miRNA and mRNA were found to be associated with nonobstructive azoospermia. The value below the nodes represents fold changes of expression levels of miRNA or mRNA between the treated and control groups.

**Figure 4:**
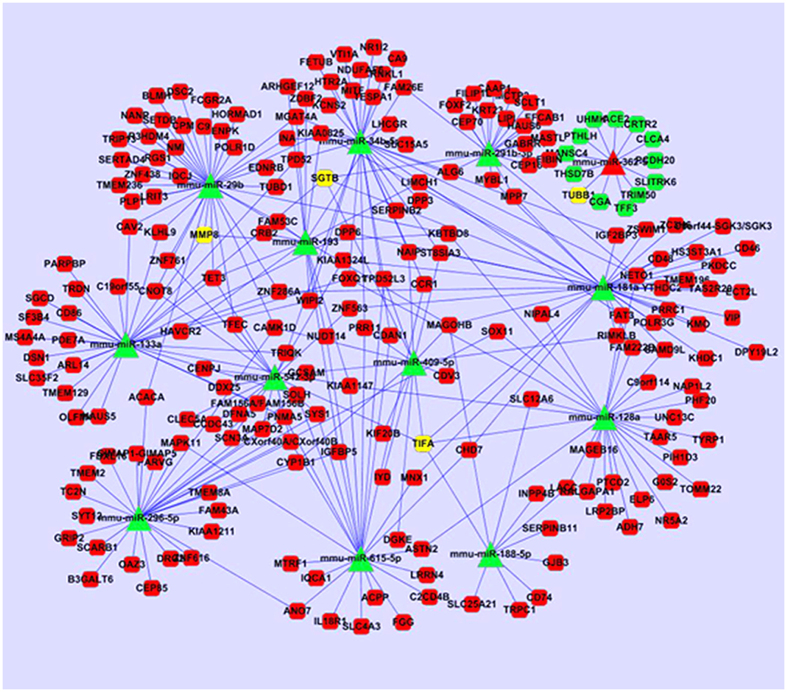
Integrated miRNA/mRNA network analysis. The differentially expressed miRNAs and target mRNAs were integrated by connecting miRNA with predicted target genes, and the regulatory networks were constructed by Cytoscape 3 software. The round rectangle nodes represent the mRNA, the triangle nodes represent the miRNA, and the red nodes represent up-regulation while the green nodes represent down-regulation. The yellow nodes represent the core gene in the sub-network.

We chose the top 20 up-regulated and top 20 down-regulated miRNAs as core miRNAs to construct miRNA regulatory networks. We predicted the targeted genes of miRNAs based on the transcriptome data. When we compared the set of predicted target genes that were under the control of those top 20 up-regulated miRNAs with the set of down-regulated genes following MC-LR treatment, 258 overlapping genes were identified in these two sets (Supplementary data 1). Likewise, we identified 439 overlapping genes in the set predicted by the top 20 down-regulated miRNA and the set containing up-regulated genes following MC-LR treatment (Supplementary data 2). With respect to the molecular functions, GO analyses revealed that these target genes may participate in cellular growth and proliferation, cell-to-cell signaling and interaction, cellular assembly and organization, and macromolecule synthesis (Fig. 1B). Interestingly, GO analyses revealed overlapping of the target genes with those of differentially expressed genes, indicating that the target gene prediction is consistent with mRNA microarray analyses. Moreover, the biological functional analyses of target genes from the Ingenuity Pathway Analyses (IPA) database demonstrated that nonobstructive azoospermia was significantly correlated with these ectopic miRNAs and mRNAs (Fig. 3). Integrative network analyses of those significantly deregulated miRNAs and mRNAs generated some functional miRNA-mRNA networks modulated by MC-LR. We also constructed a sub-network of some miRNAs (Fig. 4). TNF receptor associated factor (TRAF)-interacting protein with a forkhead-associated (FHA) domain (TIFA), small glutamine-rich tetratricopeptide repeat (TPR)-containing β (SGTB) and matrix metalloproteinase-8 (MMP-8) were found to be core miRNA-regulated genes.

### miRNA targets were involved in inducing Sertoli cell apoptosis

**Figure 5:**
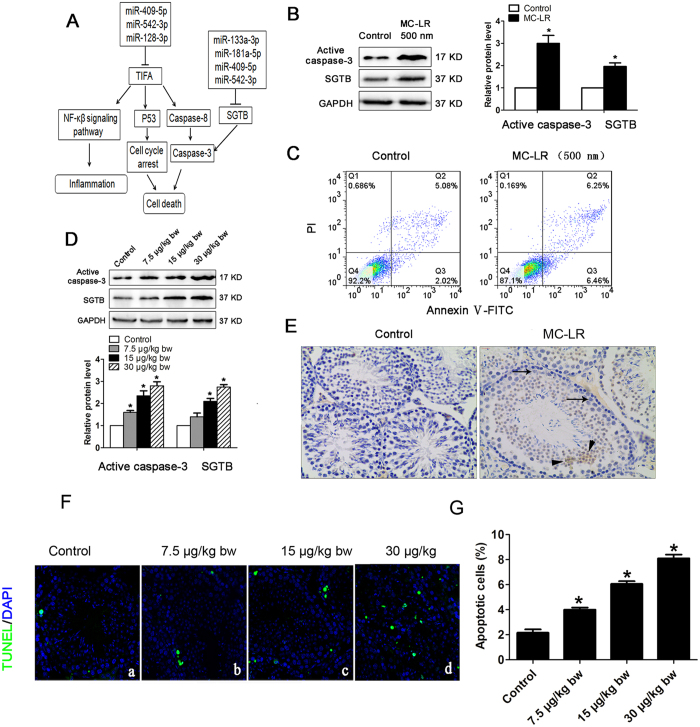
Induction of cell apoptosis by Microcystin-leucine arginine (MC-LR). (**A**) A putative model showing TNF receptor associated factor (TRAF)-interacting protein with a forkhead-associated (FHA) domain (TIFA) and small glutamine-rich tetratricopeptide repeat (TPR)-containing β (SGTB) are involved in MC-LR-induced cell apoptosis. (**B**) The protein levels of SGTB and active caspase-3 in Sertoli cells treated with MC-LR (500 nm) were measured by western blotting, and the expression levels of the proteins were quantified by densitometry and normalized to the expression of GAPDH. Densitometry data are shown as mean ± SD. *P < 0.05, compared with the control. (**C**) The number of apoptotic cells was determined by flow cytometric analysis. Cells were stained with FITC-conjugated annexin V and PI and then were analyzed by flow cytometry. (**D**) The protein levels of SGTB and caspase-3 in mouse testes were examined by western blotting, and the expression levels of the proteins were quantified by densitometry and normalized to the expression of GAPDH. Densitometry data are shown as mean ± SD. *P < 0.05, compared with the control. (**E**) Expression of active caspase-3 was examined by immunohistochemistry (×200). Arrows and arrowhead indicate Sertoli cells and germ cells. (**F**) Apoptotic cells of testicular tissues were determined by terminal deoxynucleotidyl transferase-mediated dUTP nick end labeling (TUNEL) assay (×600). Nuclei were stained with DAPI (blue) and green dots indicate apoptotic cells. (a) Control, (b) 7.5 μg/kg body weight (bw), (c) 15 μg/kg bw, (d) 30 μg/kg bw. (**G**) Apoptotic index was calculated as the percentage of TUNEL positive cells in all cells in an image (mean ± SD. *P < 0.05, compared with the control).

TIFA, with an up-regulated gene transcriptional level (Supplementary Figure S1B), was predicted to be a target gene of miR-409-5p and miR-128-3p; and these miRNAs all showed decreased expression upon MC-LR treatment (Supplementary Table S2). Up-regulated expression of TIFA may promote two independent apoptosis signaling pathways: the induction of p53 causing cell cycle arrest, and the activation of caspase-8 and caspase-3 (Fig. 5A). SGTB plays a crucial role in mediating cell apoptosis via the caspase-3-dependent pathway^29^. In this study, we also observed up-regulated expression of SGTB, targeted by miR-133a-3p, miR-181a-5p, miR-409-5p, and miR-542-3p, at both the transcriptional and protein levels in Sertoli cells following exposure to MC-LR (Supplementary Figure S1B and Fig. 5B). Interestingly, MC-LR also induced increased expression of active caspase-3 in Sertoli cells following exposure to MC-LR, which may be associated with Sertoli cell apoptosis. As predicted, flow cytometric analyses showed that MC-LR treatment induced Sertoli cell apoptosis (Fig. 5C). Moreover, up-regulated expression of SGTB was also observed in the testes of mice treated with MC-LR (Fig. 5D).

Immunohistochemical results confirmed that caspase-3 was located in Sertoli cells and germ cells in the testicular tissues of mice after intraperitoneal administration of MC-LR for 7 days (Fig. 5E). This is consistent with the analysis by terminal deoxynucleotidyl transferase-mediated dUTP nick end-labelling (TUNEL) assay demonstrating increased numbers of apoptotic cells with increasing doses of MC-LR administered (Fig. 5F,G).

### MC-LR treatment causes testicular inflammation

**Figure 6:**
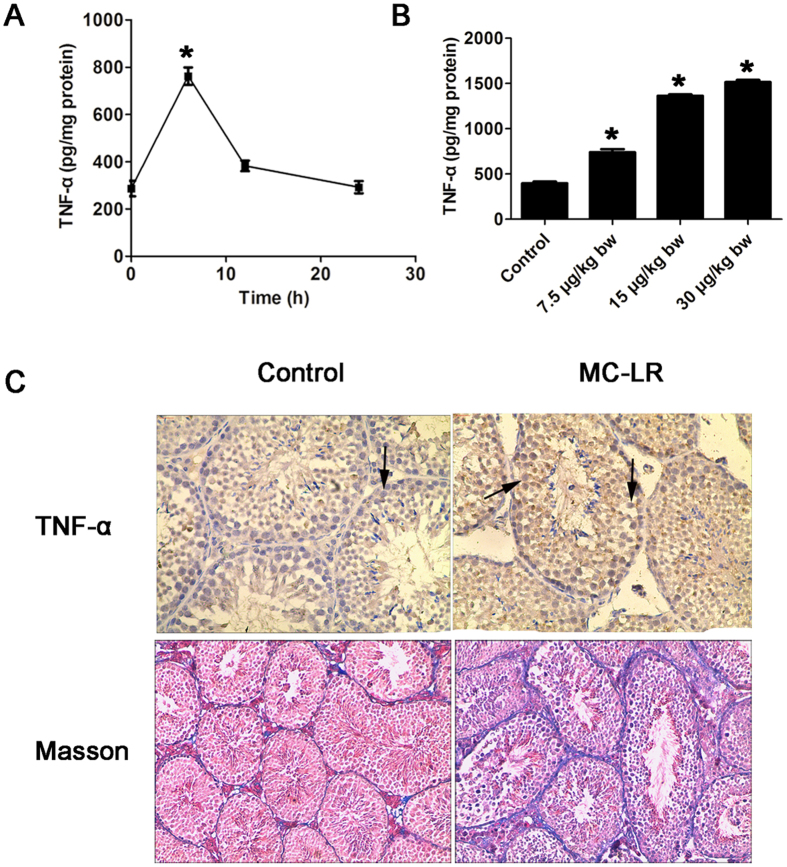
Induction of testicular inflammation by microcystin-leucine arginine (MC-LR). Sertoli cells were treated with 500 nm MC-LR for various durations (6 h, 12 h, and 24 h). (**A**) Tumor necrosis factor-α (TNF-α) levels in cells were measured with an ELISA kit (mean ± SD. *P < 0.05, compared with the control). Male mice were injected intraperitoneally with saline or MC-LR (7.5 μg/kg body weight (bw), 15 μg/kg bw, and 30 μg/kg bw) for 7 days. (**B**) TNF-α levels in testicular homogenate were determined by ELISA (mean ± SD. *P < 0.05, compared with the control). (**C**) The levels of TNF-α in testes were also examined by immunohistochemistry (×200). Arrows indicate Sertoli cells. Masson’s trichrome was used to examine the fibrosis levels in the testes after 90-day MC-LR treatment, and the presence of collagen (blue staining) was increased after MC-LR treatment (×100).

MC-LR significantly increased TNF-α expression in a time-dependent manner in cultured Sertoli cells (Fig. 6A). Levels of TNF-α peaked 6 h after treatment with MC-LR. Moreover, TNF-α was also significantly enriched in the testes of mice 7 days after intraperitoneal administration of MC-LR (Fig. 6B). Despite the fact that TNF-α was predominantly localized in Sertoli cells in the testes of control mice (Fig. 6C), substantially enhanced signals of TNF-α were detected in the testes of treated mice. Based on these results, we can confirm that MC-LR is able to induce TNF-α expression in Sertoli cells. The fibrosis levels in testicular biopsies of mice after exposure to low-dose MC-LR for 90 days were evaluated with Masson’s trichrome stain. Low-dose chronic exposure to MC-LR exhibited thickened basement membranes, suggesting enhanced fibrosis of the testicular tissue (Fig. 6C).

**Figure 7:**
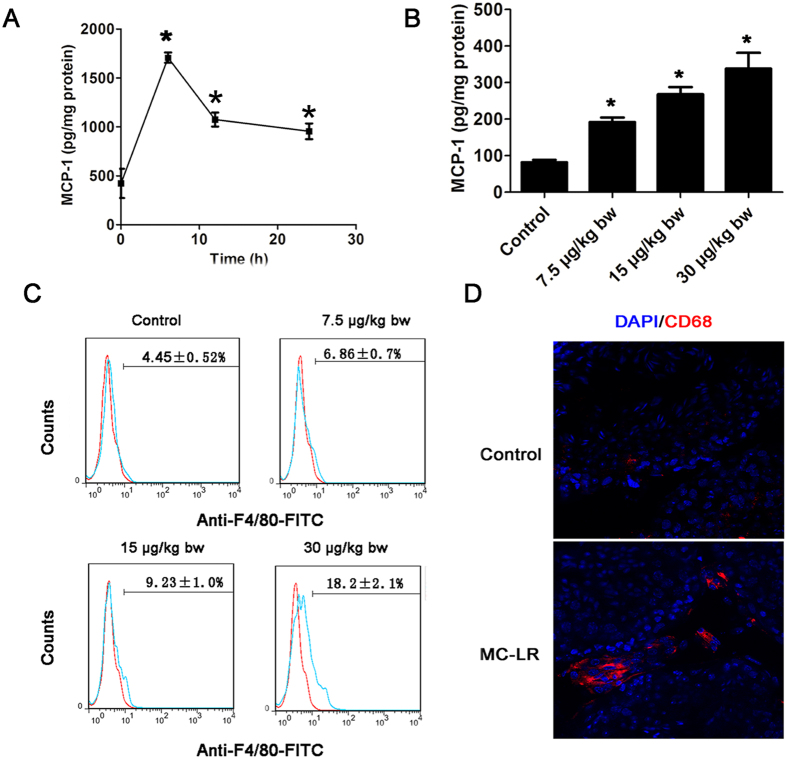
Modulation of macrophage infiltration in testicular tissues by microcystin-leucine arginine (MC-LR) treatment. Sertoli cells were treated with 500 nm MC-LR for various durations (6 h, 12 h, and 24 h). (**A**) Monocyte chemoattractant protein-1 (MCP-1) levels in cells were measured with ELISA (mean ± SD. *P < 0.05, compared with the control). (**B**) MCP-1 levels in testicular homogenate were determined by ELISA (mean ± SD. *P < 0.05, compared with the control). (**C**) Absolute number of macrophages labeled with PE-conjugated anti-F4/80 antibody was determined by flow cytometry (mean ± SD). (**D**) The distribution of macrophages labeled with CD68 (red) in the testes was examined by immunofluorescent microscopy (×600). The nuclei were stained with DAPI (blue).

Monocyte chemoattractant protein-1 (MCP-1) expression levels in MC-treated Sertoli cells and testis tissue were significantly up-regulated compared with the control group (Fig. 7A,B). Immunostaining and flow cytometric analyses demonstrated that MC-LR exposure significantly modulated macrophage numbers in the testes of the treated mice compared with controls (Fig. 7C,D).

### Integrative miRNA/mRNA analyses reveal molecular alteration associated with BTB destruction induced by MC-LR

**Figure 8:**
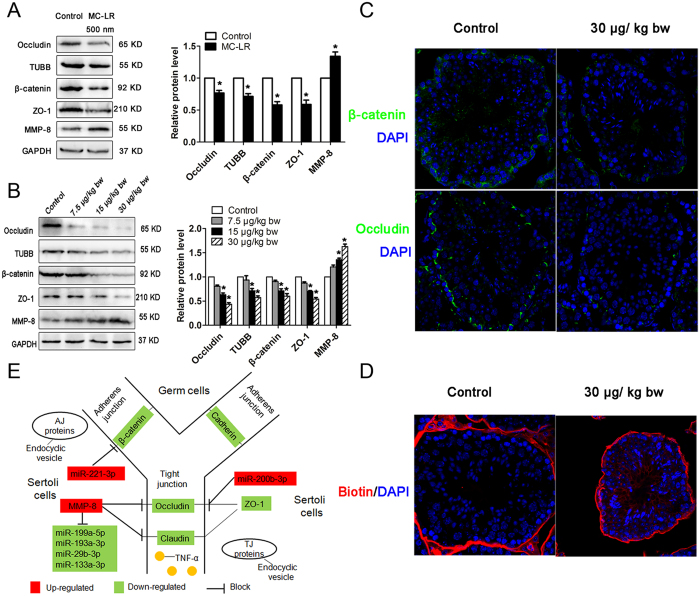
Reduced expression of tight junction (TJ) proteins and induction of blood-testis barrier (BTB) disruption by microcystin-leucine arginine (MC-LR) treatment. (**A**,**B**) Expression of occludin, ZO-1, β-catenin, TUBB and matrix metalloproteinase-8 (MMP-8) in Sertoli cells (**A**) and testes (**B**) were measured with western blotting. Expression levels of the proteins were quantified by densitometry and normalized to the expression of GAPDH. Densitometry data are shown as mean ± SD. *P < 0.05, compared with the control. (**C**) The expression of β-catenin and occludin were examined by immunofluorescent microscopy (×600). Nuclei were stained with DAPI (blue). (**D**) Impairment of BTB in mice treated with MC-LR was assessed by biotin tracer experiments. Biotin was injected underneath the testicular capsules of mice. Frozen sections were stained by Streptavidin-Alexa Fluor 594 (red) and DAPI (blue). Biotin was examined by immunofluorescent microscopy (×600). (**E**) A putative model is proposed to show the involvement of miRNAs in the Sertoli cell-Sertoli cell junction pathways.

In this study, we observed that expression levels of occludin, TUBB, β-catenin, and ZO-1 were significantly decreased in Sertoli cells following exposure to 500 nm MC-LR, suggesting damaged tight junctions between Sertoli cells (Fig. 8A). We next explored the effect of MC-LR on the BTB in testes. MC-LR significantly reduced the expression of occludin, TUBB, β-catenin, and ZO-1 assessed by western blotting (Fig. 8B) and immunofluorescence assay (Fig. 8C). BTB integrity can be evaluated by monitoring the diffusion of a biotin tracer from the interstitium to the seminiferous epithelium. In the present study, the biotin tracer was restricted to the interstitial space and the seminiferous tubule basal compartment in the saline-injected testes. In contrast, the biotin tracer was found to diffuse through the BTB into the seminiferous tubules after MC-LR treatment for 7 days, indicating disruption of BTB integrity by MC-LR (Fig. 8D). In this study, we also found that MMP-8 expression was up-regulated in Sertoli cells and testes following exposure to MC-LR (Fig. 8A,B).

### BTB breakdown promotes generation of autoantibodies against male germ cell antigens

**Figure 9:**
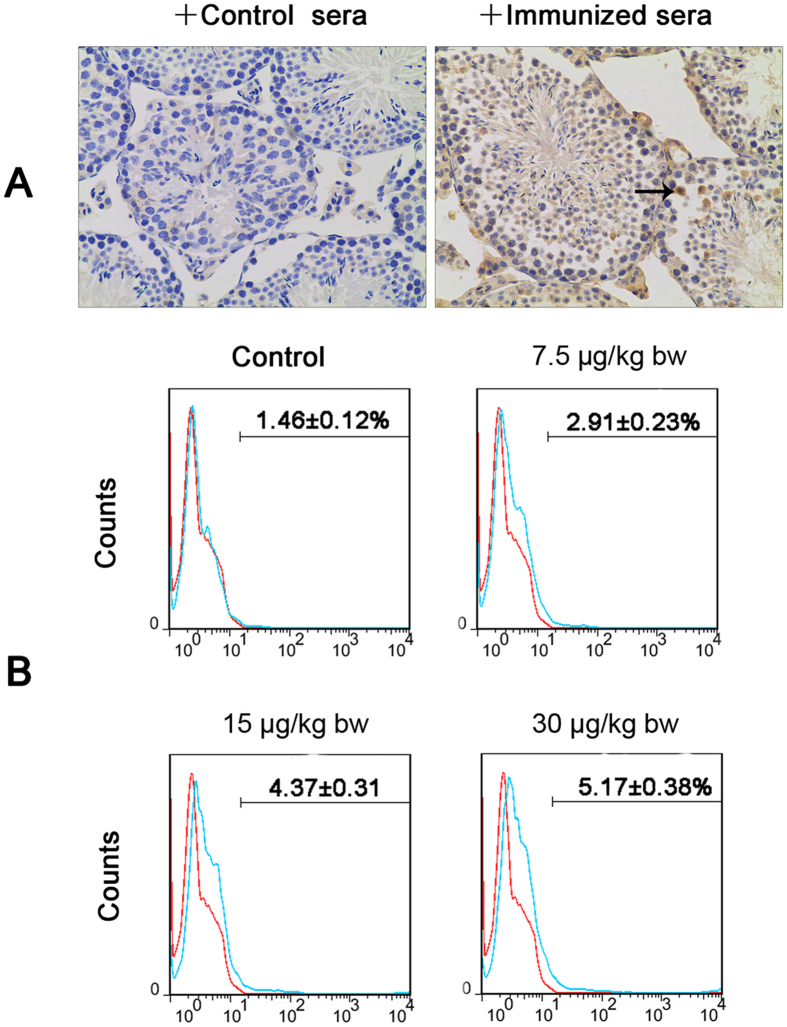
Autoantibodies production and B cells infiltration in the testes. (**A**) Production of autoantibodies against germ cells was examined by immunohistochemistry (×200). Testicular paraffin sections were immunostained with sera (1:50 dilution) of control and immunized mice that received intraperitoneal injection of MC-LR (30 μg/kg body weight) for 7 days. Arrow indicates germ cells. (**B**) The infiltration of B cells was analyzed by flow cytometric analysis. Testicular interstitial cells were labeled with FITC-conjugated anti-CD45R/B220 antibody and analyzed by flow cytometry.

Breakdown of the BTB exposes germ cells to immune surveillance, resulting in systemic autoimmunity. Sperm autoimmunity is an important contributing factor in male infertility. Sera of mice treated with MC-LR recognized male germ cells in contrast to the negative staining by the sera from control mice (Fig. 9A). These results support the generation of autoantibodies against male germ cell antigens in mice treated with MC-LR. We next analyzed the number of B lymphocyte in the testicular interstitium of control and treated mice using flow cytometry. MC-LR significantly increased B-cell numbers in the testes, which may be associated with the generation of autoantibodies against male germ cells (Fig. 9B).

### MC-LR induces TNF-α expression in Sertoli cells via the MAPK11/ATF2 signaling pathway

**Figure 10:**
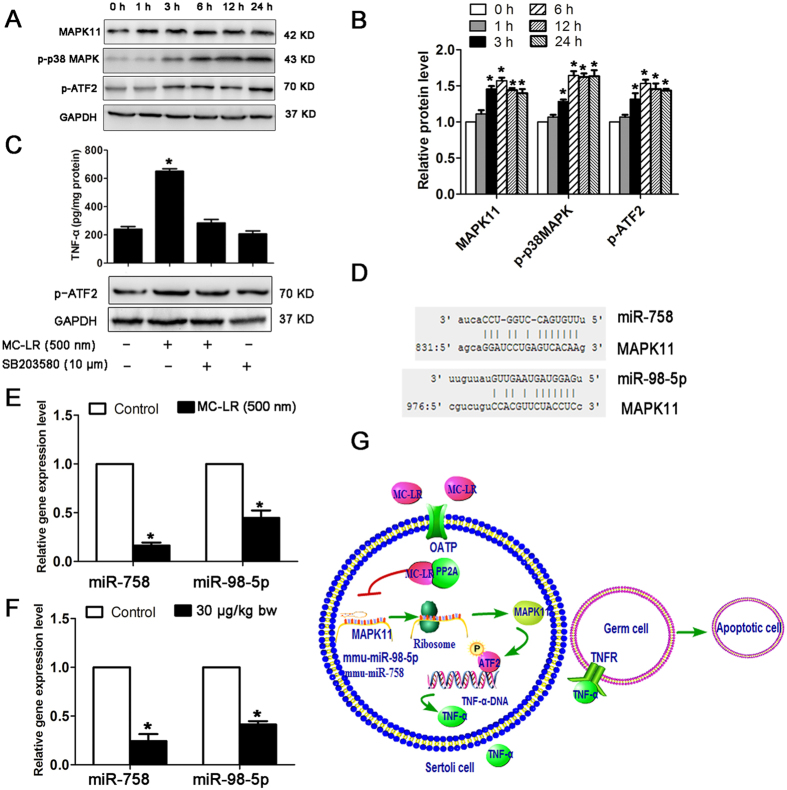
miR-98-5p and miR-758 regulatory networks involved in microcystin-leucine arginine (MC-LR)-induced tumor necrosis factor-α (TNF-α) production in Sertoli cells. (**A**) Protein levels of mitogen-activated protein kinase 11 (MAPK11, p38 β isoform), p-p38 MAPK, and p-ATF2 in Sertoli cells cultured with medium supplemented with 500 nm MC-LR for various durations were examined by western blotting. (**B**) Expression levels of the proteins were quantified by densitometry and normalized to the expression of GADH. Densitometry data are shown as mean ± SD. *P < 0.05, compared with the control. (**C**) TNF-α production in Sertoli cells treated with MC-LR or SB203580. Cells were pre-incubated with 10 μm SB203580 for 1 h, followed by another incubation with 500 nm MC-LR for 24 h. TNF-α levels in cells were measured with ELISA kits (mean ± SD. *P < 0.05, compared with the control). (**D**) Predicted binding sites of miR-98-5p and miR-758 in the MAPK11 3′-UTR region are indicated. (**E**,**F**) The expression levels of miR-758 and miR-98-5p in Sertoli cells (**E**) and testes (**F**) were measured by q-PCR, and relative mRNA abundance was calibrated based on the miRNA amount of the corresponding gene in the untreated group (mean ± SD. *P < 0.05, compared with the control). (**G**) A model for the regulation of TNF-α gene expression by miRNAs is proposed. MC-LR stimulation significantly inhibits miR-98-5p and miR-758 expression, resulting in enhanced expression of MAPK11. MAPK11 is activated by p38 signaling cascade and subsequently phosphorylates transcription factor ATF-2. Phosphorylated ATF-2 binds to the promoter of TNF-α to stimulate TNF-α expression. Secreted TNF-α can bind to the tumor necrosis factor receptor 1 (TNFR1) of the surrounding cells and induce cell apoptosis.

MC-LR treatment promoted the expression of MAPK11, p-p38 MAPK, and p-ATF2 at the protein level in Sertoli cells following exposure to MC-LR (Fig. 10A,B). Furthermore, treatment with SB203580, a selective inhibitor of p38 MAPK, significantly suppressed the activation of ATF2 and the production of TNF-α (Fig. 10C). Taken together, our data support that MC-LR stimulated TNF-α expression via promoting the MAPK11/ATF2 signaling pathway. Through integrative miRNA/mRNA expression profiling, we found that miR-758 and miR-98-5p may regulate MAPK11 expression (Fig. 10D), and decreased expression of these two miRNAs in Sertoli cells (Fig. 10E) and testes of mice (Fig. 10F) may promote MAPK11 protein expression. MAPK11 signaling increased not only the phosphorylation but also the protein levels of ATF2, thus activating the TNF-α gene transcription (Fig. 10G).

### TNF-α induces germ cells apoptosis via interacting with TNFR1

**Figure 11:**
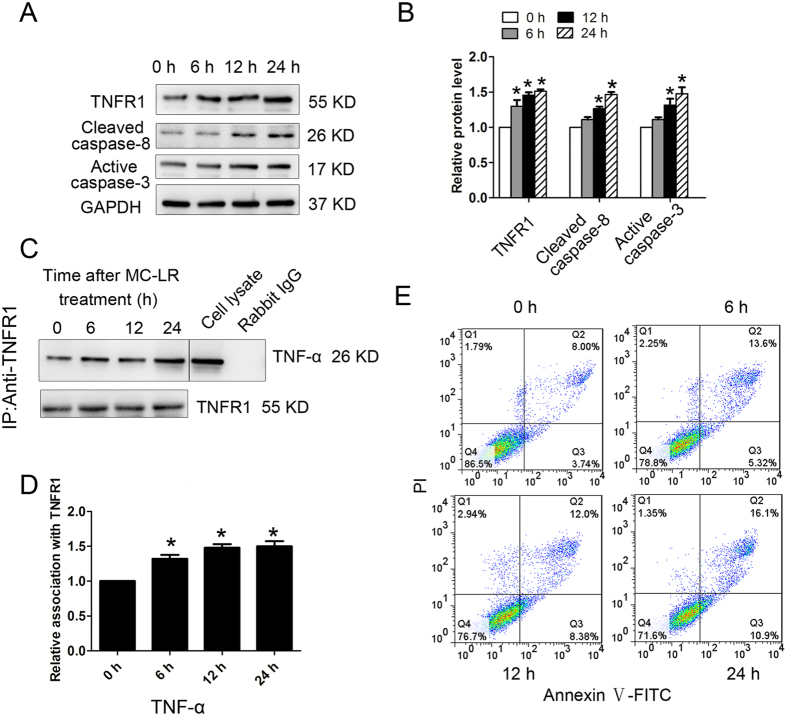
Tumor necrosis factor-α (TNF-α)-induced germ cell apoptosis via interacting with tumor necrosis factor receptor (TNFR1). Germ cells were treated with 20 ng/mL TNF-α for various durations (0 h, 6 h, 12 h, and 24 h). (**A**,**B**) The expression of TNFR1, cleaved caspase-8, and active caspase-3 in germ cells at specified time points were examined by western blotting. Representative gel electrophoresis bands are shown (**A**), and the expression levels of the proteins were quantified by densitometry and normalized to the expression of GAPDH (**B**). Densitometry data are shown as mean ± SD. *P < 0.05, compared with the control. (**C**) Germ cell lysates were subjected to coimmunoprecipitation (Co-IP) with anti-TNFR1 antibody, and the blot was probed with anti-TNF-α antibody. Moreover, blots were re-probed with anti-TNFR1 antibody to confirm equal protein loading. Co-IP with rabbit IgG served as a negative control. The presence of TNF-α in the cell lysate was detected by western blotting, serving as a positive control. (**D**) Densitometric analysis of data shown in C is plotted, and the relative association at time 0 h was arbitrarily set as 1. Data are shown as mean ± SD. *P < 0.05, compared with the control. (**E**) The number of apoptotic cells was determined by flow cytometric analysis. Cells were stained with FITC-conjugated annexin V and PI and then were analyzed by flow cytometry.

As MC-LR could stimulate the secretion of TNF-α by Sertoli cells, we were intrigued to confirm that TNF-α could bind to TNFR1 on the surrounding germ cells to induce apoptosis. To this end, mouse germ cells (GC-1 cells) were treated with TNF-α (20 ng/mL) for various durations. Furthermore, MC-LR caused a significant increase of TNFR1, cleaved caspase-8, and active caspase-3 in a time-dependent manner (Fig. 11A,B). We next performed coimmunoprecipitation (Co-IP) assay to confirm the interaction of TNF-α with TNFR1 which peaked at 6–24 h after MC-LR treatment (Fig. 11C,D), which coincided with apoptosis of GC-1 cells (Fig. 11E).

## Discussion

In this study, we analyzed the miRNA expression profile in Sertoli cells after MC-LR treatment. A set of sensitive biomarkers for monitoring and assessing the toxic effects of MC-LR on organisms can be developed based on the miRNA response profiling, which may help to predict potential MC-LR-mediated diseases. Many miRNAs associated with reproductive system diseases or tumors were found to be significantly modulated by exposure to MC-LR (Table 1). Zhao *et al*. reported that MC-LR can alter the expression of a number of miRNAs and proteins involved in several pathways related to tumorigenesis^30^. Combining these results, we infer a possible role of miRNAs in the development or establishment of MC-LR-mediated diseases. Moreover, GO enrichment analyses revealed that those differentially expressed genes in Sertoli cells following exposure to MC-LR were involved in many biological processes (Table 2). Zhao *et al*. reported several proteins regulated by MC-LR in mouse liver, which are mainly involved in metabolism, ion homeostasis, cell communication, cell binding and responses to chemical stimuli^30^. Overall, these results suggest that MC-LR exposure can cause cell death as a result of systemic dysfunction of various cellular aspects.

It is reported that the p53 signaling pathway dysfunction is evident in carcinogenesis through the regulation of a series of signal transduction pathways, resulting in the occurrence and development of a variety of malignant tumors^31^. More than 50% of tumors have been identified to involve aberrant p53 signaling pathways associated with the inactivation or mutation of the p53 protein^32^,^33^,^34^. Epidemiological studies suggest that MC-LR in drinking water is associated with a high risk for liver cancer in certain areas of China where drinking water sources are potentially contaminated with MCs^32^,^33^,^34^. Induction of the expression of proto-oncogenes is demonstrated to have tumor-promoting activity^35^. We showed here that exposure to MC-LR induced mRNA and protein synthesis of c-Fos and c-Jun, two important members of the proto-oncogene family, in Sertoli cells and testes. Taken together, this study has provided evidence that MC-LR exposure may induce carcinogenesis in the testes.

TNF-α belongs to the TNF superfamily of cytokines which play an important role in inflammation, innate and adaptive immunity, and cell death induction^36^. In the seminiferous tubules, TNF-α secreted by Sertoli cells and germ cells are capable of inducing reversible BTB disruption, through reducing the steady-state levels of occludin and ZO-1 at the BTB^37^,^38^. In this study, we not only demonstrated MC-LR-induced TNF-α expression by Sertoli cells *in vitro*, but also demonstrated TNF-α production by tissue Sertoli cells following treatment of the mice with MC-LR. Sertoli cell-derived TNF-α may possibly act on the surrounding germ cells through autocrine or paracrine modes of action. Once TNF-α binds to the TNFR1 of germ cells, the TNFR1 signaling pathway can be activated triggering germ cell death^39^, resulting in oligospermia or azoospermia in males^9^,^10^ (Fig. 10G). Indeed we observed reduced sperm concentration after MC-LR treatment (Table 3). Using Co-IP we showed the interaction of TNF-α with TNFR1 on germ cells, which may enhance the expression of active initiator caspase-8 and active caspase-3, leading to GC-1 apoptosis. However, it should be noted that Sertoli cells are not the only source of TNF-α in the seminiferous tubules. Infiltrated macrophages are also capable of synthesizing TNF-α in the testes. Moreover, we have observed TNF-α positive germ cells in the testes after MC-LR administration, suggesting one more source of TNF-α. MAPK11 can phosphorylate the transcription factor ATF2, which stimulates TNF-α expression by binding to its promoter^40^. Through integrative miRNA/mRNA expression profiling, we found that miR-758 and miR-98-5p may regulate MAPK11 expression. MAPK11 signaling increased not only the phosphorylation but also the protein levels of ATF2, thus activating the TNF-α gene transcription (Fig. 9G).

Pathway enrichment analyses showed that some hepatic fibrosis-related genes were significantly modulated by MC-LR exposure. Fibrotic thickening in the walls of the seminiferous tubules in the testes of men with impaired spermatogenesis is observed, making fibrosis a hallmark of male infertility^41^. In testes, thickened basement membranes may disrupt diffusion of nutrients into the seminiferous tubules, resulting in germ cell apoptosis and loss^41^. In this study, thickened basement membranes were observed in mice following low-dose chronic MC-LR exposure. We believe that long-term exposure to low dosage MC-LR may initiate chronic inflammation in the testes, contributing to testicular fibrosis and finally resulting in oligospermia or azoospermia in males.

For the immune system to recognize pathogens, tumor cells or dead cells and launch effector responses, leukocytes such as neutrophils, macrophages, and T-lymphocytes are recruited to the precise location of inflammation regulated by the interaction between chemokines and receptors^42^. In this study, the chemokine signaling pathway was found to be significantly enriched in the testes of mice following exposure to MC-LR. Increased numbers of macrophages were observed in the testes of the treated mice. Macrophages can secrete a variety of proinflammatory cytokines (e.g. TNF-α, IL-6, IL-1β) that may induce testicular tissue damage, germ cell apoptosis and BTB disruption^36^,^38^,^43^,^44^,^45^. MCP-1 is an important chemokine that can attract immune effector cells to the injured sites to regulate many physiological processes such as development, wound repair, and immunity^46^. Significant enrichment of MCP-1 in the testes of treated mice can promote immune cell infiltration into the testes. However, there was not significant change of transcriptional level of MCP-1, which is not consistent with the protein measurement. This discrepancy may be attributed to the post-transcriptional regulation of protein expression.

In mammals, the BTB is formed by adjacent Sertoli cells in the seminiferous epithelium near the basement membrane via the coexisting specialized TJ, basal ectoplasmic specialization (a testis-specific atypical adherens junction), and the desmosome-like junction^15^. Several integral membrane protein complexes constitute the BTB in mammal testes, such as the occludin-ZO-1 complex at the TJ and the N-cadherin-β-catenin complex at the basal ectoplasmic specialization. In mammals, the BTB confers immune privilege upon maturing germ cells, whose disruption may lead to the production of anti-sperm antibodies, causing immunological infertility. Exposure to environmental toxicants such as cadmium, mercury, bisphenol A can lead to male infertility through the disruption of TJ as a result of redistribution of junction proteins (e.g. occludin, N-cadherin) from the cell-cell interface to the cytosol^47^,^48^,^49^. In this study, expression levels of occludin, TUBB, β-catenin, and ZO-1 were significantly decreased in Sertoli cells and the testes of mice following exposure to MC-LR; the biotin tracer assay showed that MC-LR can disrupt BTB integrity. These data support that MC-LR can perturb spermatogenesis by destroying the microenvironment of spermatogenesis.

Microtubules, located in the peripheral areas of Sertoli cells, can promote the successful transit of spermatogenic cells from the basal compartment to the apical compartment^50^. The absence of normal microtubule organization can lead to the failure of spermatogenic cell differentiation and development^51^. In Sertoli cells treated with MC-LR, the expression of TUBB targeted by miR-362-5p was significantly decreased, implicating compromised spermatogenesis by MC-LR through impeding germ cell transit from the basal compartment to the tubular lumen. AJ proteins (e.g. E-cadherin, β-catenin) are present in the seminiferous tubules to adhere germ cells onto the Sertoli cells, and their destruction causes loss of germ cells from the seminiferous epithelium^52^. Exposure to MC-LR may cause the internalization of AJ proteins; germ cells are eventually released from the seminiferous epithelium prematurely as a result of disruption of adhesion complexes (Fig. 8E). Exposure to MC-LR decreased the sperm concentration compared, which may be attributed to the release of germs from seminiferous epithelium and germ cell apoptosis (Table 3).

As a core target gene of miRNAs, MMP-8 can degrade extracellular matrix proteins, and their aberrant expression may cause breakdown of the blood-brain barrier (BBB) inducing infiltration of peripheral immune cells, resulting in induction of neuroinflammation^53^,^54^. miR-193, miR-29b and miR-133a were predicted to regulate MMP-8 expression at the translational level, and a significantly down-regulated expression of these miRNAs was observed in MC-treated Sertoli cells, which may therefore promote MMP-8 expression.

Developing male germ cells produce abundant immunogenic autoantigens, which do not induce detrimental immune responses in the testes under physiological conditions because of the testicular immunoprivileged properties^44^. Once the testicular immune privilege is disrupted, immune responses against autoantigens are initiated, thereby perturbing male fertility. Generation of autoantibodies against male germ cell antigens in the sera might be due to the disruption of the BTB induced by MC-LR.

In summary, our data confirmed a miRNA-mRNA integrated network in Sertoli cells following exposure to MC-LR. Cytotoxicity induced by MC-LR may be subject to the regulation of TIFA, SGTB, MMP-8, Occludin, TUBB1, β-catenin, MAPK11, and TNFR1. The clinical relevance of these specific signature miRNAs and their targeting pairs still need further validation. Our work has laid a foundation for identifying potential molecular markers for the diagnosis and treatment of the related diseases.

## Materials and Methods

### Ethics statement

The animal experiments were performed according to the Guide for the Care and Use of Laboratory Animals (The Ministry of Science and Technology of China, 2006) and all experimental protocols were approved under the animal protocol number SYXK (Su) 2009–0017 by the Animal Care and Use Committee of Nanjing University.

### Main chemicals

MC-LR was purchased from Alexis Biochemicals (Lausen, Switzerland). Fetal bovine serum (FBS), Dulbecco’s Modified Eagle’s Medium-Ham’s F-12 Nutrient Mixture (DMEM-F12) was obtained from Gibco (Grand Island, NY). Collagenase I, trypsin, penicillin, and streptomycin sulphate were purchased from Sigma-Aldrich (St. Louis, MO). Specifications of primary antibodies used for different experiments were listed in Supplementary Table S3. TNF-α and MCP-1 ELISA kits were obtained from eBioscience (San Diego, CA). Annexin V-FITC apoptosis detection kit was purchased from BD bioscience (San Jose, CA).

### Primary cell culture and MC-LR treatment

Sertoli cells were isolated and cultured as previously reported^11^,^55^. Testes from 5 mice (20-day-old) were aseptically removed, decapsulated, minced, and washed twice with sterile PBS. The minced tissues were then digested with 0.25% trypsin at 37 °C for 6 min, followed by digestion in 0.1% collagenase I at 37 °C for 10 min. Next, the homogenate obtained was filtered through a 150-μm filter, and cells were collected by centrifugation at 300 × *g* for 5 min. After being washed with PBS for 3 times, the isolated Sertoli cells were re-suspended in culture medium containing 90% DMEM-F12 medium and 10% FBS and then plated on cell culture dishes. Cells were maintained in a humidified atmosphere of 95% air/5% CO2 (v/v) at 37 °C. Sertoli cells were adherent to the bottom of the dishes after culture for 2 days. Next, these cultures were subjected to a hypotonic treatment to lyse residual germ cells^15^,^55^. After 2 to 3 days, these cells formed a monolayer. The expression of marker proteins (AR, SOX9, Nr5a1, and DMRT1) was confirmed by immunofluorescence staining to identify the purity of cultured Sertoli cells.

### miRNA and mRNA expression by microarray analysis

The comparative analysis of microarray data between treated samples and control samples was carried out using independent *t* test. False discover rate (FDR) was further controlled by the adjustment of *P* value using Benjamini-Hochberg algorithm. All statistical tests and visualization of differentially expressed genes were done within R environment^56^. miRNAs with *P* < 0.05 and fold change ≥ 2 and ≤ 0.5 were determined to be statistically significant. Using miRNA microarray, we have previously identified 46 miRNAs that were significantly up-regulated (FDR < 0.05) and 69 miRNAs that were down-regulated (FDR < 0.05) in Sertoli cells following exposure to MC-LR for 24 h (Supplementary Table S1)^20^. The raw mRNA expression data were deposited in the Gene Expression Omnibus Database (GEO, https://www.ncbi.nlm.nih.gov/geo/) under accession number GSE87475. For analyzing mRNA microarray data, we would have obtained too few differentially expressed genes if we used a 2.0 fold-change as cutoff. In order not to lose any potential genes that were associated with the cytotoxicity induced by MC-LR, we chose to set 1.2 as the fold-change cutoff. A total of 2494 genes were differentially expressed in Sertoli cells after treatment with MC-LR, including 1037 up-regulated genes (fold change ≥ 1.20) and 1457 down-regulated genes (fold change ≤ 0.83)^20^.

### q-PCR validation analyses of miRNAs and target genes

Total RNA from the testes and Sertoli cells was isolated using Trizol reagent (Invitrogen). The purity of RNA was determined with a spectrophotometer (Hoefer, Holliston, MA). To examine the mRNA levels, one μg of total RNA was then reversely transcribed using a first strand cDNA synthesis kit (Takara, Dalian, China). q-PCR was conducted using the SYBR Green qRT-PCR kit (Takara) on a ViiA 7 Q-PCR System (Applied Biosystems, Waltham, MA). The amplification efficiency of each pair of primers was tested by constructing the corresponding plasmid; only primers with a similar amplification efficiency were used in this experiment (Supplementary Table S4). The q-PCR was carried out in a 10 μl reaction mixture, which consisted of 5 μl of 2 × SYBR Green I Mix, 0.4 μl of forward and reverse primers (10 μM of each primer), and 4.2 μl diluted cDNA. Cycling conditions were as follows: Denaturation at 95 °C for 30 s, followed by 40 cycles of denaturation for 5 s at 95 °C, annealing for 30 s at 60 °C and extension for 30 s at 72 °C. Expression levels of 10 miRNAs randomly chosen was examined with the Taqman miRNA assay kit (Invitrogen) according to manufacturer’s instruction (Supplementary Table S2). The relative quantification values for each miRNA and mRNA were calculated by the 2^−ΔΔct^ method using U6 and GAPDH as internal references, respectively. Based on the fold changes of miRNA assessed by miRNA microarray or q-PCR, we obtained normalized miRNA expression values by calculating the logarithmic value of fold changes. We performed correlation analysis for the two sets of data.

### miRNA-mRNA regulatory network construction

TargetScan (http://www.targetscan.org/), miRBase (http://www.mirbase.org/), and miRanda (http://www.microrna.org/microrna/home.do) were used to predict the target genes of differentially expressed miRNAs. The predicted target genes were compared with the mRNA microarray results; only genes with an expression pattern that negatively correlated with its regulatory miRNAs were included. Cytoscape software (Version 3.0; http://www.cytoscape.org/) was used to construct the regulatory network^57^. Moreover, miRNA regulatory network analyses was performed using IPA to describe functional relationships among miRNAs and genes based on the known associations in the databases (IPA^®^, QIAGEN Redwood City, www.qiagen.com/ingenuity), which mapped those differentially expressed genes to biological pathways^58^.

### Gene ontology and pathway enrichment analyses of differentially expressed genes

To identify potential biological processes regulated by the differentially expressed genes, we performed GO and pathway enrichment analyses based on the GO (http://geneontology.org/page/go-enrichment-analyses) and pathways (http://www.genome.jp/kegg/pathway.html) database. We calculated the *P* value of each GO term using right-sided hypergeometric tests, and Benjamini-Hochberg adjustment was used for multiple test correction^59^,^60^. We consider those terms with a *P* value < 0.05 as significantly enriched terms.

### Animals and treatment

Male specific pathogen-free (SPF) BALB/c mice aged six weeks were purchased from Laboratory Animal Centre of the Academy of Military Medical Science, China. Mice were randomly divided into 4 groups: one control and three experimental groups with 20 mice in each group. Mice in the experimental group were injected intraperitoneally with MC-LR dissolved in saline at 7.5 μg/kg, 15 μg/kg and 30 μg/kg body weight for 7 consecutive days; the control mice were treated with identical volumes of normal saline. One day after the last treatment, mice were sacrificed by CO_2_ asphyxiation and testes were obtained for analyses of histopathology, q-PCR, and western blotting. In separate experiments, we investigated the toxic effects of chronic low-dose exposure to MC-LR on the male reproductive system. Mice were divided into 5 groups with 20 mice in each. For exposure, mice were given drinking water containing 1 μg/L, 10 μg/L, 20 μg/L, or 30 μg/L MC-LR for 90 consecutive days. Control mice were provided with only the blank water. Mice were sacrificed later and testes were obtained for histopathological analyses.

### TNF-α-induced germ cell apoptosis

In order to confirm the effect of TNF-α on germ cell apoptosis, mouse GC-1 cells that share many of the characteristics of spermatogonia were treated with 20 ng/mL TNF-α *in vitro* for 3 h, 6 h, or 12 h. Cell apoptosis was measured by flow cytometry based on Annexin V –FITC and PI staining profiles. Protein was extracted from both control and treated cells for further analyses.

### Western blotting, Co-IP and ELISA analyses

One hundred milligrams of tissue were homogenized for extracting proteins in 1 mL ice-cold RIPA buffer (50 mM Tris-HCL PH 7.4, 150 mM NaCl, 1% NP, 0.1% SDS, 1 × phosphatase/protease inhibitors). For some cells, 200 μl protein extraction buffer was added into the dish to extract the total protein from cells. The protein concentration was determined using the BCA protein assay kit (Beyotime, Nantong, China). Western blotting was performed as described previously^13^. About 20 μg of protein from each sample was separated on 10% SDS-PAGE and electrophoretically transferred to a polyvinylidene fluoride (PVDF) membrane. The membrane was then blocked in TBS buffer containing 5% bovine serum albumin (20 mM Tris–HCl (pH 7.6) and 150 mM NaCl) for 1.5 h at 37 °C. Next, these blots were incubated overnight at 4 °C with rabbit anti-c-Fos, rabbit anti-c-Jun, rabbit anti-SGTB, rabbit anti-β-catenin, rabbit anti-MAK11, rabbit anti-Occludin, rabbit anti-ZO-1, rabbit anti-p-p38 MAPK, rabbit anti-active caspase-3, rabbit anti-p-ATF2, goat anti-ATF2, mouse anti-caspase-8, and mouse anti-TNFR1. The blots that were incubated with rabbit IgG, mouse IgG, and goat IgG, served as negative controls. The blots were then incubated with species-matched horseradish peroxidase-conjugated secondary antibodies (Boster, Wuhan, China). The chromogenic signal intensity was detected using an Odyssey Scanning System (LI-COR, Lincoln, NE) and quantified using image J software (NIH, Bethesda, MD). Co-IP was performed by using 800 μg of protein from lysates of GC-1. Anti-TNFR1 antibody was used as the precipitating antibody to isolate TNFR1 from lysates, followed by western blotting to identify whether TNF-α can bind to TNFR1 on the membrane using anti-TNF-α antibody. Blots were re-probed with anti-TNFR1 antibody to confirm equal protein loading. Co-IP with rabbit IgG served as a negative control. TNF-α and MCP-1 levels in cell supernatant and testis homogenate were measured by ELISA kits (eBioscience, San Diego, CA). All protocols were performed according to the manufacturer’s instructions. Detection limits of both kits were <10 pg/mL, intra- and inter- assay variation was <5% and <10%, respectively.

### Immunohistochemistry, Masson’s trichrome stain and TUNEL assay

Testes were fixed in 4% paraformaldehyde solution for 24 h, embedded in paraffin and cut into 5-μm-thick sections. The sections were incubated with PBS containing 3% H_2_O_2_ for 10 min to inhibit endogenous peroxidase activity. After being blocked with 3% BSA in PBS for 1 h at 37 °C, the sections were subsequently incubated with rabbit anti-TNF-α and rabbit anti-active caspase-3 antibodies overnight at 4 °C and then with HRP-conjugated secondary antibodies (Boster) at 37 °C for 1 h. HRP activity was examined using the diaminobenzidine method according to the manufacturer’s instructions (Zhongshan Biotechnology, Beijing, China). After counterstaining with haematoxylin, the sections were mounted with neutral balsam for observation under a microscope DXM12000F (Nikon, Tokyo, Japan). Masson’s trichrome stain was used to determine the collagen deposits in the testes according to the instructions by the manufacturer (KeyGen, Nanjing, China). Apoptotic cells in testicular tissues were determined by TUNEL assay as described by the manufacturer (Roche Applied Science, Mannheim, Germany), and the nuclei were stained with DAPI (Sigma-Aldrich). The images were captured using a laser scanning confocal fluorescence microscope (FV10i; Olympus, Tokyo, Japan). At least 20 microscopic fields (×600) randomly selected were evaluated for each testis. MC-LR-induced apoptotic index was calculated as the percentage of TUNEL positive cells in all cells in an image.

### Immunofluorescence staining

Immunofluorescence analyses of testicular tissues were performed as previously described^12^. The following primary antibodies were employed: Rabbit anti-β-catenin, rabbit anti-occludin, and mouse anti-CD68. Alexa Fluor 488-conjugated goat anti-rabbit antibody or Alexa Fluor 594-conjugated goat anti-mouse antibody (Invitrogen) was used as a secondary antibody.

### Biotin-tracing assay

Freshly dissected testes were injected with 10 mg/mL EZ-Link Sulfo-NHS-LC-LC-Biotin (Thermo, Waltham, MA) dissolved in PBS. After being incubated at 37 °C for 30 min, the testes were subsequently fixed in 4% paraformaldehyde for 4 h, followed by being immersed in a 30% sucrose solution. The specimens were embedded in optimal cutting temperature (OCT, Sakura Finetek USA, Torrance, CA) and then were cut at a thickness of 8 μm followed by fixation in acetone at 4 °C for 15 min. The sections were blocked with 3% BSA to avoid non-specific staining prior to incubation with Streptavidin-Alexa Fluor 594 (Invitrogen). After nuclear staining with DAPI, sections were observed under the laser scanning confocal fluorescence microscope FV10i.

### Flow cytometric analyses

Flow cytometric analyses were used to determine whether immune cells were enriched in testicular tissues. The testes were de-capsulated in PBS and then gently pipetted to dissociate the seminiferous tubules, resulting in the release of interstitial cells. Cell suspensions were passed through 100-μm copper meshes to remove the seminiferous tubules, after which the interstitial cells were collected by centrifugation by 300 × *g* for 5 min. Cells were incubated with PE-conjugated F4/80 antibodies or FITC-conjugated CD45R/B220 (BD Biosciences). The cells were analyzed using a FACScallbur flow cytometer (BD Biosciences). Cell apoptosis was analyzed by an Annexin V-FITC and PI staining kit (Vazyme, Nanjing, China) according to manufacturer’s instructions.

### Statistical analyses

SPSS 18.0 (SPSS, Chicago, IL) was used for statistical analysis. Experimental results were expressed as mean ± standard deviation. The Levene’s test were employed to check the normality and homogeneity of variances in the data. The Student’s *t* test was used for paired comparisons. For the comparison of three or more groups, one-way ANOVA was used for the comparison, which was followed by Duncan’s *post hoc* test. Values of *P < *0.05 were considered statistically significant.

## Additional Information

**How to cite this article**: Chen, Y. *et al*. Microcystin-Leucine Arginine Causes Cytotoxic Effects in Sertoli Cells Resulting in Reproductive Dysfunction in Male Mice. *Sci. Rep.* **6**, 39238; doi: 10.1038/srep39238 (2016).

**Publisher’s note:** Springer Nature remains neutral with regard to jurisdictional claims in published maps and institutional affiliations.

## Supplementary Material

Supplementary Dataset 1

Supplementary Dataset 2

## Figures and Tables

**Table 1:** List of miRNAs associated with infertility and cancer in the integrated network.

Functional Annotation	miRNA
nonobstructive azoospermia	miR-128-3p,miR-181a-5p, miR-199a-5p, miR-29b-3p miR-34a-5p, miR-362-5p, miR-374-5p, miR-450a-5p
urinary tract tumor	miR-133a-3p, miR-138-5p, miR-199a-5p, miR-29b-3p
prostate cancer	miR-29b-3p, miR-34a-5p
genital tumor	miR-29b-3p, miR-34a-5p
cancer	miR-125a-3p, miR-128-3p, miR-133a-3p, miR-181a-5p, miR-188-5p, miR-193a-3p, miR-199a-5p, miR-296-5p, miR-342-5p, miR-34a-5p miR-362-5p, miR-374b-5p, miR-423-5p, miR-450a-5p, miR-542-3p, miR-671-3p, miR-7a-5p

**Table 2:** Target enrichment in distinct pathways.

Ingenuity Canonical pathway	*P* value	Ratio
coagulation System	4.47E-04	1.32E-01
T helper cell differentiation	3.54E-03	6.94E-02
chemokine signaling	4.0E-03	5.00E-02
TNFR1 signaling	4.1E-03	2.31E-01
inducible Nitric Oxide synthetase signaling	4.9E-03	2.31E-01
Sertoli cell-Sertoli cell junction signaling	4.5E-03	2.04E-02
p53 signaling	4.8E-03	2.08E-01
antigen presentation pathway	8.9E-03	5.00E-01
hepatic fibrosis activation	4.8E-03	2.05E-01
bladder cancer signaling	5.0E-03	2.17E-01

**Table 3:** Quantitative analysis of sperm quality from mice treated with MC-LR.

Groups	Epididymidal sperm concentration (×10^6 ^mL^−1^)	Sperm motility (%)	Sperm abnormality (%)
0 μg/(kg day) (n = 10)	21.65 ± 3.45	62.54 ± 8.82	8.26 ± 0.88
7.5 μg/(kg day) (n = 10)	17.22 ± 2.24*^a^	50.48 ± 6.26*^a^	13.68 ± 2.12*^a^
15 μg/(kg day) (n = 10)	14.18 ± 1.26*^b^	34.26 ± 4.18*^b^	18.88 ± 2.68*^b^
30 μg/(kg day) (n = 10)	10.56 ± 1.42*^c^	25.16 ± 4.22*^c^	26.26 ± 4.22*^c^

Data are shown as mean ± SD. Superscript letters (a–c) denote variances that are significantly different from each group (*P* < 0.05). *Response that is significantly different from the control (*P* < 0.05).
